# Examining the autistic traits in children and adolescents diagnosed with attention-deficit hyperactivity disorder and their parents

**DOI:** 10.1186/s12888-020-02703-z

**Published:** 2020-06-05

**Authors:** Esra Okyar, Işık Görker

**Affiliations:** 1 Department of Child and Adolescent Psychiatry, Sakarya University Training and Research Hospital, Sakarya, Turkey; 2 Department of Child and Adolescent Psychiatry, Faculty of Medicine, Trakya University, Edirne, Turkey

**Keywords:** ADHD, Autism, Autistic traits, Parents, Child

## Abstract

**Background:**

Attention-Deficit Hyperactivity Disorder (ADHD) and Autism Spectrum Disorder (ASD) are two of the most frequently-observed neurodevelopmental disorders. Autistic traits are detected frequently in children who have ADHD. This study aimed to examine autism symptoms in children diagnosed with ADHD and their parents; and also, to investigate parental risk factors that increase autistic traits in children. Besides the risk factors related to pregnancy, birth and developmental history were examined.

**Methods:**

Two groups were created consisting of 66 children diagnosed with ADHD and 33 children not diagnosed with ADHD and their parents. Autism symptoms were screened with the Autism Behavior Checklist (ABC) in children, and Autism Spectrum Quotient (AQ) in parents. Also, Adult ADD/ADHD DSM-IV Based Diagnostic Screening and Rating Scale and Wender Utah Rating Scale (WURS) were used to determine ADHD symptoms in parents.

**Results:**

It was determined that there were more autism symptoms in children who were diagnosed with ADHD than in the control group without ADHD. There were more autistic symptoms in boys and the presence of Oppositional Defiant Disorder (ODD). Although there were more ADHD symptoms in the parents of children diagnosed with ADHD, it was determined that they did not differ from parents in the control group in terms of autism symptoms. It was also determined that maternal and paternal ADHD symptoms were predictive for autism symptoms in children. It was also shown that maternal smoking during pregnancy is associated with more autistic traits.

**Conclusion:**

ASD and ADHD show high levels of comorbidity. The etiology remains unclear. Both ADHD and ASD show strong hereditary transition. We found that maternal and paternal ADHD symptoms predict autism symptoms in children with ADHD. However, more studies are needed to reveal the etiology.

## Background

Attention-Deficit Hyperactivity Disorder (ADHD) and Autism Spectrum Disorder (ASD) are two of the most frequent neurodevelopmental disorders detected in children [1]. The prevalence of ASD is 1%, while the prevalence of ADHD is approximately 5% [2]. Both disorders have attention problems, difficulty in peer communication, impulsivity and varying degrees of hyperactivity, which cause important behavioral, academic, emotional and adaptive problems at school, home and other environments [3]. Both disorders are seen more frequently in boys [4]. ADHD accompanies ASD at a rate of 22–83%; and ASD accompanies ADHD at a rate of 30–65% [2]. The comorbidity of ADHD and ASD was associated with lower quality of life [5].

The diagnostic criteria of DSM-IV-TR would not allow the diagnosis of ASD and ADHD together in the same case, which caused that ASD and ADHD were examined separately for some time. Recent studies have revealed that ADHD symptoms accompany autism, and autism symptoms accompany ADHD frequently, and there are clinical, genetic and neuropsychological overlapping points between autism and ADHD [6]. In this way, both ASD and ADHD were defined under the heading of Neurodevelopmental Disorders with DSM 5; and the presence of autism was eliminated from the diagnosis of ADHD [7]. Problems like inability in social communication, misinterpretation of social hints, difficulty in understanding the feelings and thoughts of others, repetitive movements, executive dysfunctions, emotion regulation problems, and delays in language development are observed frequently in ADHD and autism [8, 9].

ADHD and ASD show a high level of inherited transition. We have very little knowledge of the reasons for the relation between ADHD and ASD. According to the results of studies conducted on genetics for this subject, it is considered that the same genetic areas might be influenced by the autism and ADHD comorbidity [10]. In a twin study, autistic symptoms were detected in children who were diagnosed with ADHD, and it was found that more autistic symptoms accompanied the combined type of ADHD [11]. In another twin study, more autism symptoms were detected in the siblings of children who were diagnosed with ADHD than non-ADHD controls, and it was observed that stereotypic movements were higher at a significant level [8]. According to the results of a twin study conducted on young adults, autism and ADHD symptoms tend to be seen together [12].

The results differ in studies that investigate the relation between ADHD subtype and autistic symptoms. In some studies, autistic symptoms were determined to be more frequent in combined type ADHD; in some other studies, no significant differences were detected between ADHD subgroups [13]. Although more aggression and intrusive behaviors in social communication are seen in combined type ADHD, in the type in which attention-deficit is dominant, poor performance in games is more common due to introvert symptoms and poor memory [14].

ADHD shows a strong familial transition. While the incidence of ADHD being detected in at least one child of the ADHD-diagnosed parents is 50%; the family of a child diagnosed with ADHD being affected by ADHD is 25% [15]. According to the results of a study conducted with children and mothers diagnosed with ASD or ADHD, the risk of ADHD was six-fold higher in the first child of a mother diagnosed with ADHD, and the risk of ASD increased 2.5-fold [1]. In another study in which the parents of children diagnosed with ADHD and children not diagnosed with ADHD were included, it was determined that both parents of children diagnosed with ADHD had more psychiatric symptoms and more ADHD symptoms than the parents of the control group [16].

In a study that investigated the psychopathologies of families of children diagnosed with ADHD, significant relations were detected between adult-onset psychiatric disorders (psychotic disorders, bipolar disorder, depression, anxiety disorder, personality disorders and substance abuse) and childhood ADHD; however, no relations were detected between childhood-onset psychiatric disorders (autism, communication disorder, learning disorder and intellectual disability). The mother’s having ADHD showed a stronger relationship than the father’s having ADHD [17].

The diagnosis of ADHD might delay the diagnosis of ASD. In a study conducted with 1496 children diagnosed with ASD, it was shown that nearly 20% of the cases were diagnosed with ASD after being diagnosed with ADHD. It was determined that those diagnosed with ADHD were diagnosed with ASD after 3 years on average [18]. In another study conducted in Taiwan, similarly, ADHD diagnosis was found to delay the diagnosis of ASD [19]. For this reason, the diagnosis of ASD must also be considered in children who have ADHD symptoms [18].

The aim of the present study was to examine the autistic traits of children diagnosed with ADHD and their parents, and to determine whether there is a relation between autistic traits in children and autism symptoms in parents. In addition, risk factors increasing autism symptoms related to pregnancy, birth and developmental history were also examined.

## Methods

### Design

The present study is a descriptive study in which autistic traits and ADHD symptoms are investigated with appropriate scales in children, adolescents and parents.

### Participants

A total of 66 ADHD patients (16 girls and 50 boys, aged 6–15 years) and their parents who applied to Trakya University Medical Faculty Child and Adolescent Psychiatry Clinic between November 2017–May 2018 were included in our study as ADHD group; and 33 children (9 girls and 24 boys, aged 6–15 years) without ADHD diagnosis and their parents were included as the control group. A detailed psychiatric interview was made with all participants by the researcher. The diagnosis of ADHD was made according to the Diagnostic and Statistical Manual of Mental Disorders Fifth Edition (DSM 5), and ASD was excluded according to DSM 5. Children with a total ABC score of 68 or more have a high probability of being autistic. Therefore, they were excluded from the study. Intellectual functioning was assessed with the Wechsler Intelligence Scale for Children-Revised (WISC-R). Participants whose total Intelligence Quotient (IQ) score were above 80 were included in the study. Having important medical, neurological and genetic diseases in both groups and having bipolar disorder, psychosis, important medical, neurological and genetic diseases in the parents were determined as exclusion criteria. Since the present study was a family study, the children who were adopted were not included in the study.

ADHD group cases met the diagnostic criteria with 34% attention-deficit predominant type ADHD, 12% hyperactivity / impulsivity predominant type ADHD, and 54% combined ADHD. Three cases in girls and 14 cases in males were diagnosed with ODD and one case was diagnosed with Conduct Disorder according to DSM-IV-Based Child and Adolescent Behavior Disorders Screening and Rating Scale.

The mean age of the patients in the ADHD group was 10.1 ± 2.5 years and, the mean age of the control group was 10.0 ± 2.8 years. There was no statistically significant difference between the ADHD group and the control group in terms of the mean age (*p* > 0.05) and gender (*p* = 0.744). In the ADHD group, the education level of the parents and monthly income were found to be significantly lower than in the control group (*p* < 0.005) (Table 1).

**Table 1:** Comparison of sociodemographic characteristics and pregnancy and birth history of ADHD group and control group

	ADHD Group (***n*** = 66)		Control Group(***n*** = 33)		***p*** Value
	Number	Percentage	Number	Percentage	
**Gender**					0.744
Girl	16	%24.2	9	%27.3	
Boy	50	%75.8	24	%72.7	
**Child Education Level**					0.653
Primary School	34	%51.5	16	%48.5	
Secondary School	25	%37.9	15	%45.5	
High School	7	%10.6	2	%6.0	
**Mother Education Level**					0.021*
Primary School	18	%27.3	5	%15.2	
Secondary School	9	%13.6	2	%6.0	
High School	22	%33.3	7	%21.2	
University	17	%25.8	19	%57.6	
**Father Education Level**					0.001*
Primary School	14	%21.2	3	%9.1	
Secondary School	8	%12.1	4	%12.1	
High School	26	%39.4	4	%12.1	
University	18	%27.3	22	%66.7	
**Monthly Income**					< 0.001*
< 1500 Turkish liras	5	%7.6	0	%0.0	
1500–3499 Turkish liras	42	%63.6	8	%24.2	
3500–5000 Turkish liras	6	%9.1	11	%33.3	
> 5000 Turkish liras	13	%19.7	14	%42.5	
**Smoking in Pregnancy**					0.400
No	56	%84.8	33	%90.9	
Yes	10	%15.2	3	%9.1	
**Problem in Pregnancy**					0.428
No	57	%86.4	31	%93.9	
Yes	9	%13.6	2	%6.1	
**Birth Type**					0.196
Normal	25	%37.9	17	%51.5	
C-Section	41	%62.1	16	%48.5	
**Low Birth Weight**					0.579
No	64	%97.0	31	%93.9	
Yes	2	%3.0	2	%6.1	
**Prematurity**					0.138
No	58	%88	32	%97	
Yes	8	%12	1	%3	
**Problem at Birth**					< 0.001*
No	38	%57.6	33	%100	
Yes	28	%42.4	0	%0	

*p < 0.05

No problem at birth was detected in the control group. In the ADHD group, 4 cases had asphyxia, 1 case had a traumatic delivery, 1 case had premature rupture of membranes, 1 case had an infection, 20 cases had jaundice, and 1 case had asphyxia and jaundice. When ADHD and control groups were compared in terms of problems at birth, there were significantly more problems in the ADHD group compared to the control group (*p* < 0.05).

### Measurements

A sociodemographic data form was applied to all participants. The ages, genders, educational status, parents’ ages, parents’ educational levels, parents’ jobs, family structure, monthly income, prenatal, natal and postnatal development history, psychiatric diagnosis and treatment status were questioned with this form.

#### DSM-IV-based child and adolescent behavior disorders screening and rating scale

The scale has 41-items, and was developed by transforming the diagnostic criteria of DSM-IV into questions without changing their meanings. It was developed by Atilla Turgay. There are 9 questions investigating attention-deficit in the scale, 6 questions investigating hyperactivity, 3 questions investigating impulsivity, 8 questions investigating Oppositional Defiant Disorder (ODD), and 15 items investigating Behavioral Disorders. The scale is filled by the mothers, fathers and teachers of children, who are suspected of having ADHD. Each item is rated between 0 and 3 (0 = none, 1 = slightly, 2 = more, 3 = too much). For ADHD diagnosis, at least 6 of the 9 items investigating attention-deficit or hyperactivity and impulsivity must be rated as 2 or 3 [20].

#### Autism behavior checklist (ABC)

This is a family report scale consisting of a total of 57 items and five sub-scales (sensory, relating, body and object use, language and social skills) [21]. It is used to determine the autistic symptom levels in the child. The validity and reliability study of the scale was conducted for Turkish with 3–15 year old individuals. The lowest score that may be received from the scale is 0, and the highest score is 159. If the score received from the scale is high, this shows that the autistic symptoms are more [22].

#### Adult ADD/ADHD DSM-IV based diagnostic screening and rating scale

The original English version was developed by Atilla Turgay. It is in the form of a self-report scale to screen attention-deficit, hyperactivity and impulsivity in adults according to DSM-IV diagnostic criteria. It consists of 48 items and 3 subscales rated between 0 and 3. The attention-deficit section consists of 9 items, the hyperactivity/impulsivity section consists of 9 items, and the ADHD-related characteristics section consists of 30 items on the scale. In the attention-deficit or hyperactivity/impulsivity section, it is accepted that there is a low-level attention-deficit or hyperactivity/impulsivity symptom in those who receive scores below 3; there is a moderate-level attention deficit or hyperactivity/impulsivity symptom in those who receive a score between 3.01 and 10.99; and there is a high-level attention deficit or hyperactivity/impulsivity symptom in those who receive scores above 11. Those who receive scores between 0 and 12.99 in the ADHD-related behavior section have a low-level, those who receive scores between 13 and 35 have moderate-level, and those who receive a score between 35 and 75 have high-level characteristics that are stated in the scale. Those who score less than 20 on the entire scale show low ADHD symptoms; those who score between 20 and 59 show moderate ADHD symptoms; and those who score more than 59 show high ADHD symptoms. The Turkish validity and reliability study was conducted by Günay et al. [23].

#### Wender Utah rating scale (WURS)

This scale questions ADHD symptoms in childhood retrospectively, and consists of 25 items and five subscales [24]. It is a five-point Likert-type scale and each item is scored between 0 and 4. Item 8 is scored reversely. The total score of the scale is obtained by collecting the points from all items. According to the scale score, adults are not diagnosed. In Turkish validity and reliability study, 36 points have been determined as cut-off points [25].

#### Autism Spectrum quotient (AQ)

It is a self-report scale of 50 items measuring autistic features in individuals with normal intelligence, and was developed by Baron Cohen. It consists of 5 subscales. Each of the subscales (social skill, attention switching, attention to detail, communication and imagination) consists of ten items. The increase in the total score indicates that the autistic symptoms are high. In its Turkish validity and reliability study, the cut-off point was found 26 [26].

### Statistical analyses

Mean, standard deviation, median, frequency and ratio values were used in descriptive statistics of the data. The Kruskal-Wallis and Mann-Whitney U and Wilcoxon tests were used to analyze the quantitative data. In the analysis of the qualitative independent data, the Chi-square test was used. Spearman correlation analysis was employed for correlation analysis. Statistical Package for the Social Science (SPSS) 22.0 database program was used in the analyses. The statistical level of significance was determined as *p* ≤ 0.05.

## Results

### Developmental history

In the ADHD group, time to start sitting was 6.3 ± 1.2 months and in the control group it was 6.3 ± 0.7 months. Time to start walking was 12.3 ± 2.1 months in the ADHD group, and 12.5 ± 1.9 months in the control group. The mean time for saying the first words was 12.33 ± 4.5 months in the ADHD group, and 11.7 ± 4.5 in the control group. The time to start forming two-word sentences was 22.2 ± 4.9 months in the ADHD group and, 17.1 ± 4.6 months in the control group. When the ADHD group and the control group were compared regarding the development history, there was no significant difference between the two groups in terms of sitting, walking and saying the first words. (*p* > 0.05). It was observed that the ADHD group started later to establish a two-word sentence compared to the control group (*p* < 0.001).

### Comparison of ABC scores of ADHD and non ADHD groups

When the ADHD group and the control group were compared in terms of ABC scores, the ADHD group’s all subscale scores and total scores were found significantly higher than those of the control group (Table 2). In the ADHD group, ABC total scores were found significantly higher in boys than in girls (*p* = 0.027). In the presence of ODD, ABC scores were determined higher (*p* = 0.001). There was no significant difference between ADHD subgroups (attention-deficit predominant, hyperactivity/impulsivity predominant and combined type), and ABC sensory, relating, body and object use and language subscale scores and the total score (*p* > 0.05). Only in the combined ADHD subgroup, the ABC social skills subscale score was significantly higher than attention-deficit predominant and hyperactivity/impulsivity predominant type (*p* = 0.02).

**Table 2:** Distribution of ABC subscale and total scores of cases

	ADHD Group		Control Group		***p*** Value
	Mean ***±*** SD	Median	Mean ***±*** SD	Median	
**Sensory**	2.7 ± 3.5	1.5	0.8 ± 1.9	0.0	0.001*
**Relating**	8.5 ± 6.5	7.5	1.3 ± 2.7	0.0	< 0.001*
**Body and Object Use**	5.2 ± 5.7	3.0	0.9 ± 2.4	0.0	< 0.001*
**Language**	4.5 ± 4.1	3.5	0.7 ± 2.4	0.0	< 0.001*
**Social Skills**	6.3 ± 4.4	6.0	1.0 ± 3.1	0.0	< 0.001*
**Total Score**	27.1 ± 17.8	25.0	4.7 ± 9.8	0.0	< 0.001*

*p < 0.05

*SD* Standard Deviation, *ABC* Autism Behavior Checklist, *ADHD* Attention-Deficit Hyperactivity Disorder

### Analyzing parental adult ADHD scale, WURS and AQ scores

The ADHD group mothers’ WURS inattentiveness, irritability, depression and school problems subscales and total scores were found significantly higher than those of the control group. The behavioral problems/impulsiveness subscale scores were determined similarly in the mothers of both groups (*p* > 0.05). The ADHD group fathers’ WURS inattentiveness, irritability, depression and behavioral problems/impulsiveness subscale scores and total scores were significantly higher than those of the control group. In the ADHD and control group, fathers’ WURS school problems scores did not differ significantly (*p* > 0.05). The Adult ADHD scale, attention-deficit, hyperactivity/impulsivity and associated features subscale scores and total scores of the mothers in the ADHD group were found to be statistically higher than the mothers in the control group. In the Adult ADHD scale, attention-deficit, hyperactivity/impulsivity and associated features subscale scores and total scores of the fathers in the ADHD group were found to be significantly higher than the fathers in the control group. The total AQ score of the mothers in the ADHD group was found to be 19.9 ± 4.4, and the total AQ score of the mothers in the control group was 19.9 ± 4.9. While the total AQ score of the fathers in the ADHD group was 20.9 ± 4.7, total AQ score of the control group fathers were 21.1 ± 4.5. No statistically significant difference was determined between the AQ subscales and total scores of the parents in both groups (*p* > 0.05) (Table 3).

**Table 3:** Comparison of the ADHD and control group parents with WURS, Adult ADHD Scale, AQ scores

	ADHD		Control Group		***p*** Value
	Mean ***±*** SD	Median	Mean ***±*** SD	Median	
**Mother WURS**					
Inattentiveness	5.0 ± 3.5	4.0	3.1 ± 2.2	2.0	0.002*
Irritability	3.1 ± 3.9	2.0	1.4 ± 1.9	1.0	0.018*
Depression	3.1 ± 3.5	2.0	1.8 ± 2.4	1.0	0.036*
Behavioral problems/impulsiveness	1.2 ± 2.0	0.5	0.7 ± 1.3	0.0	0.178
School Problems	2.1 ± 2.3	2.0	1.0 ± 1.4	0.0	0.015*
Total	14.5 ± 12.4	11.0	8.0 ± 7.6	6.0	< 0.001*
**Father WURS**					
Inattentiveness	6.3 ± 3.3	6.0	3.5 ± 1.8	4.0	< 0.001*
Irritability	5.5 ± 6.1	3.0	2.8 ± 3.5	2.0	0.030*
Depression	3.8 ± 3.7	3.0	1.6 ± 1.8	1.0	0.001*
Behavioral problems/impulsiveness	3.1 ± 4.3	1.0	1.2 ± 2.0	0.0	0.011*
School Problems	2.2 ± 2.2	2.0	1.4 ± 1.5	1.0	0.090
Total	20.8 ± 16.3	15.5	10.5 ± 8.5	8.0	< 0.001*
**Mother Adult ADHD Scale**					
Attention Deficit	5.5 ***±*** 3.6	5.0	3.0 ± 2.9	2.0	< 0.001*
Hyperactivity/Impulsivity	4.4 ± 4.7	3.0	2.5 ± 2.5	2.0	0.046*
Associated Features	17.5 ± 12.8	13.0	9.3 ± 8.2	7.0	0.002*
Total	27.4 ± 18.6	23.0	14.8 ± 12.0	12.0	< 0.001*
**Father Adult ADHD Scale**					
Attention Deficit	5.4 ± 4.2	4.0	3.0 ± 3.5	1.0	0.001*
Hyperactivity/Impulsivity	5.0 ± 4.5	4.0	2.4 ± 3.0	2.0	0.002*
Associated Features	16.8 ± 12.1	14.0	8.1 ± 8.0	6.0	< 0.001*
Total	27.2 ± 19.3	20.0	13.5 ± 12.6	8.0	< 0.001*
**Mother AQ**					
Social Skill	3.9 ± 1.9	4.0	4.4 ± 1.8	5.0	0.172
Attention Switching	4.0 ± 1.5	4.0	3.9 ± 1.3	4.0	0.976
Attention to Detail	5.2 ± 2.3	5.0	4.5 ± 2.0	4.0	0.277
Communication	2.6 ± 1.6	2.0	2.5 ± 1.3	2.0	0.814
Imagination	4.2 ± 1.9	4.0	4.5 ± 1.8	5.0	0.399
Total	19.9 ± 4.4	20.0	19.9 ± 4.9	21.0	0.812
**Father AQ**					
Social Skill	4.2 ± 2.1	4.0	4.2 ± 1.9	4.0	0.961
Attention Switching	3.9 ± 1.6	4.0	4.5 ± 1.1	4.0	0.061
Attention to Detail	5.3 ± 1.8	5.0	4.8 ± 1.9	5.0	0.259
Communication	3.2 ± 1.5	3.0	2.6 ± 1.3	2.0	0.077
Imagination	4.3 ± 1.6	5.0	5.0 ± 1.7	5.0	0.060
Total	20.9 ± 4.7	21.0	21.1 ± 4.5	22.0	0.752

*p < 0.05

*ADHD* Attention-Deficit Hyperactivity Disorder, *SD* Standard Deviation, *WURS* Wender Utah Rating Scale, *AQ* Autism Spectrum Quotient

### Association between parental ADHD and autism symptoms and autism symptoms in children

The symptoms of ADHD in parents and autistic symptoms in children were examined by Spearman correlation analysis. Although there was no correlation between the mothers with only attention-deficit and only hyperactivity/impulsivity symptoms and ABC, a significant correlation between ADHD total score and ABC was found. A correlation was observed between the attention-deficit, hyperactivity/impulsivity, and total scores of the Adult ADHD Scale of the fathers and ABC. A significant relationship was detected between the WURS and ABC. No relation was determined between the AQ and ABC (*p* > 0.05) (Table 4).

**Table 4:** Parental correlation of parents’ Adult ADHD Scale, WURS and AQ scores with children’s ABC scores

ABC	Rho (r)	***p*** Value
**ADHD-Attention-deficit**		
Mother	0.143	0.157
Father	0.220	0.028*
**ADHD-Hyperactivity/Impulsivity**		
Mother	0.157	0.121
Father	0.346	< 0.001*
**ADHD-Total**		
Mother	0.223	0.026*
Father	0.329	0.001*
**WURS**		
Mother	0.265	0.008*
Father	0.356	< 0.001*
**AQ**		
Mother	0.070	0.494
Father	0.046	0.653

*p < 0.05

*ADHD* Attention-Deficit Hyperactivity Disorder, *WURS* Wender Utah Rating Scale, *AQ* Autism Spectrum Quotient

### Relationship between ABC scores and prenatal, natal, postnatal factors

In both groups, the ABC total score was significantly higher in the children of mothers who smoked during pregnancy than the children of non-smokers (*p* = 0.031). There was no relationship between infection, drug use and having an illness in pregnancy and ABC scores (*p* > 0.05). The mean total ABC score was found to be higher in children who had problems at birth. No correlation was found between low birth weight, prematurity, and total ABC score (Table 5).

**Table 5:** Analysis of the relationship among prenatal, natal and postnatal factors and ABC total scores

ABC-Total			
	Mean ***±*** SD	Median	***p*** Value
**Smoking in pregnancy**			0.031*
Yes	30.54 ± 20.333	30.0	
No	18.00 ± 18.122	13.50	
**Infection in pregnancy**			0.359
Yes	12.40 ± 16.517	4.00	
No	20.03 ± 18.918	14.50	
**Drug use in pregnancy**			0.605
Yes	20.13 ± 14.769	22.00	
No	19.60 ± 19.184	14.00	
**Ilness in pregnancy**			0.674
Yes	16.91 ± 17.874	8.00	
No	19.99 ± 18.987	14.50	
**Problem at birth**			0.001*
Yes	28.25 ± 16.480	28.00	
No	16.25 ±18.681	7.00	
**Low birth weight**			0.401
Yes	13.50 ± 20.339	5.50	
No	19.91 ± 18.809	15.00	
**Prematurity**			0.247
Yes	23.00 ± 11.446	20.00	
No	19.31 ± 19.399	13.50	

*p < 0.05

*SD* Standard Deviation, *ABC* Autism Behavior Checklist

## Discussion

In our study, we used ABC to screen the autism symptoms in children. All subscale and total scores of ABC were higher in the ADHD group. In the ADHD group, the total ABC score was found to be higher in boys than in girls. As a result of our study, it was determined that there are more autism symptoms in children with ADHD. This result is consistent with other studies investigating autism symptoms in children with ADHD [4, 8, 27, 28]. In this study, no relationship was found between the ADHD subgroups and ABC total score. Only in the combined ADHD subgroup, the ABC social skill subscale score was significantly higher than the attention-deficit predominant and hyperactivity/impulsivity predominant type. When the literature was reviewed, autism symptoms were determined more in combined-type ADHD; however some studies reported that there were no significant differences in ADHD subgroups in terms of autism symptoms [13]. In the ADHD group, the education level of the parents and monthly income were found to be significantly lower than in the control group. Low socio-economic status of the family increased the rates of the inattentive and hyperactive/impulsive symptoms. The psychosocial risk factors may increase ADHD symptoms in children with ASD [29]. Also, in one study, familial risk factors were found to be predictive of more ASD symptoms in children with ADHD [30].

Autism symptoms are seen more in ADHD children accompanied by ODD [30]. In our study, the ABC total score of the cases with ADHD and ODD comorbidity was found to be higher than the cases diagnosed with only ADHD. An increase in autistic symptoms was observed in the presence of ODD. Social difficulties increase in children with the comorbidity of ADHD and ODD [13]. In a study by Mulligan et al. [31], it was shown that the children who had ADHD with comorbid ODD, Social Communication Disorder, language and motor disorders had autism symptoms at higher levels. Some opposing behaviors are thought to be related to ADHD, whereas some of them are thought to be related primarily to ASD due to resistance to changes [32].

Specific speech and language problems are common in ASD and ADHD. Delay in speech development, which is one of the defining features of autism, is common in individuals with ADHD [4]. In addition, children with ADHD experience difficulties in pragmatic use of language, as do children with autism [3]. However, these difficulties in the use of language are not as severe as in children with autism. In our study, delay in speech development was observed in children with ADHD. ABC language subscale scores were also significantly higher in the ADHD group. These results confirm that children with ADHD have more problems in language development and in the use of language. The results of adult studies investigating the relationship between pragmatic language problems and ADHD symptoms were found to be consistent with the studies conducted on children. In an adult study of ADHD symptoms assessed by the Adult ADHD Self-Report Scale (ASRS), and autism symptoms assessed by the Broad Autism Phenotype Scale (BAPS), a strong correlation was found between ADHD characteristics and the pragmatic language problems subscale [33].

ADHD and autism are two neurodevelopmental disorders with a high hereditary transition. Some studies have reported that ADHD and autism pass from mother to child, and some studies have reported they pass from the father. Fewer studies have found no evidence of inheritance from mother or father [2]. A total of %33 of ADHD children had a parent with ADHD symptom presence [34]. In our study, more ADHD symptoms were found in the families of children with ADHD compared to the control group parents, which was in line with the literature. In the WURS, which retrospectively questions the ADHD symptoms of childhood, the parents of children with ADHD had higher scores than the parents of the control group. In the Adult ADHD Scale, the parents of children with ADHD are reported with a higher level of ADHD symptoms than the parents of control group. It has been reported that the parental psychiatric disorder, comorbid disorders, and severe ADHD are associated with the persistence of childhood ADHD in adulthood [35]. The presence of parental ADHD was associated with increased ADHD symptoms/behavioral problems and lower treatment response in the child [15, 34]. It has been reported that parental ADHD treatment increases parental skills, increases the participation of parents in the treatment of the child, and decreases the ADHD symptoms of the child [15].

In our study, autistic traits in parents were examined with AQ which is a scale that investigates the broad autism phenotype characteristics. In our study, no significant difference was found between the ADHD and control group parents in terms of autistic traits. In a study conducted with 334 subjects examining adult population autism and ADHD symptoms, a significant correlation was found between ADHD symptoms and autism symptoms. A relationship was determined between attention-deficit and high total ADHD scores and deficits in communication and social skills [33].

As a result of the Spearman correlation analysis, it was found that there was a significant correlation among parental attention-deficit and hyperactivity symptoms and autism symptoms in children. No correlation was determined between the AQ and ABC. According to these results, it is thought that maternal and paternal ADHD symptoms predict the autism symptoms of the child. The finding that ADHD symptoms in parents are associated with autism symptoms in children is consistent with the results of similar studies in the literature. In a study conducted with the families of 121 children diagnosed with autism, or autism and ADHD, autism and ADHD scores of the parents showed a significant correlation. A strong relationship was found between the autism scores of the mother and father and the autism symptoms of the child. It was determined that autism symptoms in parents do not predict the symptoms of ADHD in the child, but the ADHD symptoms in the mother predict autism symptoms in the child [2]. In another study, ADHD symptoms in the mother or father were associated with autism symptoms in the child. Although the autism symptoms of the mother were found to be risk factors for autism symptoms in children with ADHD, no relationship was found between the autism symptoms of the father and the autism symptoms of the child. This result suggested that autistic features are inherited by maternal transition such as Fragile X Syndrome and duplication in chromosome 15q11–13 [1]. In our study, the hypothesis that autistic traits are more prevalent in the families of the ADHD group could not be supported. This may be due to the small number of sampling. It is thought that more studies should be done with larger sampling groups. In addition, self-survey questionnaires were used in our study. Families with children with ADHD may have responded more positively about their problem behaviors. Therefore, it is thought that autism symptoms in parents should be investigated with structured psychiatric interviews.

The effect of prenatal and postnatal risk factors on autistic symptoms in ADHD patients was investigated. It was found that autism symptoms are higher in children whose mothers smoke during pregnancy. Researches show that maternal smoking during pregnancy, prenatal exposure to alcohol, young maternal age and maternal stress increase the risk for ADHD in children [36]. In an ASD-ADHD association study, it was stated that there is a gene-environment interaction for autism symptoms in children with ADHD. The interaction of maternal smoking with 5-HTLPR S / S genotype has been shown to be related to the social communicative problems. Interaction with the COMT Val / Val genotype was found to be associated with the stereotypic movements [37]. In our study, no relationship was found among pregnancy complications, prematurity and low birth weight and autism symptoms. However, a relationship was determined among complicated birth and postpartum problems and autism symptoms. Autism symptoms of children with traumatic birth, asphyxia, infection or jaundice history were found to be higher. In this respect, Rommelse et al. [38] reported that complicated pregnancy, complicated birth, low birth weight, prematurity and the low Apgar score could be a risk factor for both ADHD and ASD; and therefore, could not be helpful for differential diagnosis.

### Strengths and limitations

There are some limitations to our study. The low number of sampling is thought to limit the generalizability of the results. Also, there was a gender imbalance between the participants. It is known that clinical applications are more frequent in boys with ADHD, therefore; this may be the reason. The second limitation is that all ADHD cases were under medical treatment. Treatment may have effects on ADHD symptoms and, on autism symptoms, and the severity of symptoms. The third limitation is that the data about the children were obtained from the families, mostly from the mothers. The fourth limitation is that the ADHD and autistic traits in parents were evaluated by self-report scales in our study. Due to the lack of structured psychiatric interviews, it was interpreted whether there was evidence for ADHD and autism. It was not diagnostic. The final limitation is that there were multiple comparisons that made statistical management difficult. There are also some strengths in our study. Our study was a family study involving fathers; however, mostly, only mothers were included since there were difficulties in the participation of fathers in the family studies [39]. Also, fathers do not often participate in parenting interventions [40]. Therefore, the inclusion of fathers is the powerful aspect of our study.

## Conclusions

In summary, it was found in the present study that children with ADHD had more autistic traits than non-ADHD controls. There was a correlation among the autism symptoms in children and parental ADHD symptoms. No significant difference was found between the parents of children with ADHD and parents of the control group in terms of autism symptoms. A relationship was determined between autistic traits in children and maternal smoking during pregnancy and the presence of natal and postnatal problems. It is considered that determining the relation between ADHD and autistic traits will help identify the difficulties that might be experienced in the social and communicative field and organize treatment plans for this condition.

## Data Availability

The datasets used and/or analyzed during the current study are available from the corresponding author on reasonable request.

## Abbreviations

ABC: Autism Behavior Checklist
ADHD: Attention-deficit Hyperactivity Disorder
ASD: Autism Spectrum Disorder
AQ: Autism Spectrum Quotient
DSM-5: Diagnostic and Statistical Manual of Mental Disorders 5
IQ: Intelligence Quotient
ODD: Oppositional Defiant Disorder
SD: Standard Deviation
SPSS: Statistical Package for the Social Science
WISC-R: Wechsler Intelligence Scale for Children-Revised
WURS: Wender Utah Rating Scale
